# Case report: PSMA PET/CT addresses the correct diagnosis in a patient with metastatic prostate cancer despite negative core biopsies and mpMRI. A diagnostic challenge

**DOI:** 10.3389/fonc.2023.1101221

**Published:** 2023-02-07

**Authors:** Luigia Vetrone, Giulia Cuzzani, Riccardo Mei, Lucia Zanoni, Alessandro Bertaccini, Lorenzo Bianchi, Paolo Castellucci, Caterina Gaudiano, Alberta Cappelli, Francesca Giunchi, Stefano Fanti

**Affiliations:** ^1^ Nuclear Medicine, Alma Mater Studiorum University of Bologna, Bologna, Italy; ^2^ Nuclear Medicine, IRCCS Azienda Ospedaliero-Universitaria di Bologna, Bologna, Italy; ^3^ Division of Urology, IRCCS Azienda Ospedaliero-Universitaria di Bologna, Bologna, Italy; ^4^ Department of Radiology, IRCCS Azienda Ospedaliero-Universitaria di Bologna, Bologna, Italy; ^5^ Pathology Unit, IRRCS Azienda Ospedaliero-Universitaria di Bologna, Bologna, Italy

**Keywords:** [^68^Ga]Ga-PSMA-PET/CT, prostate cancer, staging, mini-invasive biopsy, metastatic lymph node

## Abstract

This is a case of [^68^ Ga]Ga-Prostate-specific membrane antigen (PSMA)-11 PET/CT in a 73-years old patient presenting high Prostate Specific Antigen (PSA) levels despite both multi-parametric magnetic resonance imaging (mpMRI) and 12-core saturation biopsy negative for prostate cancer (Pca). This is a highly interesting case because, despite the advanced metastatic spread at initial presentation as showed by [^68^Ga]Ga-PSMA-PET/CT, the primary Pca was detected by none of the diagnostic techniques (12 random sample biopsy, mpMRI, PSMA PET/CT). However, [^68^Ga]Ga-PSMA-PET/CT showed a suspicious axillary lesion suitable for biopsy, which finally resulted as Pca metastasis. This case report is therefore a brilliant example of how [^68^Ga]Ga-PSMA-PET/CT optimized patient’s management.

## 1 Introduction

PSMA is a trans-membrane bound glycoprotein highly expressed in several tissues such as prostate, kidney and salivary glands (1) and represents a successful target for imaging in Nuclear Medicine. PSMA overexpression is present in over 90% of Pca cells, making PSMA a reliable tissue biomarker (2, 3). PSMA binding tracers labelled with [^68^Ga] or [^18^F] are currently used in staging PCa and in restaging patients with biochemical relapse. Definitive diagnosis of Pca relies on histopathology; according to EAU guidelines (4) biopsy is performed in patients who present elevated prostate-specific antigen (PSA) screening indicators (i.e. PSA>4 ng/mL) and in those with prostate nodules detected through MRI or abnormal digital rectal examination (DRE) suggestive for clinically significant Pca.

We present the [^68^Ga]Ga-PSMA-11 PET/CT scan performed in a 73-year-old man after the detection of elevated PSA levels (doubling-time PSA<4 months) and enlarged pelvic and retroperitoneal lymphadenopathies at ultrasound, despite prostate gland negativity at both mpMRI and 12-core saturation biopsy. Nevertheless, [^68^Ga]Ga-PSMA-11 PET/CT was performed due to its good sensitivity and specificity in staging Pca patients (77% and 97% respectively for lymph node involvement (5)).

## 2 Case description

A 73-year-old man underwent a routinary abdomen ultrasound detecting pelvic and retro-peritoneal adenomegalies, confirmed by a subsequent CT scan. In addition, a clinical suspicion of Pca was raised by significantly increased PSA levels (55 ng/dl versus 2.1 ng/dl the previous year), and a saturation biopsy was performed; however, none of the 12 samples were positive for Pca. The transrectal ultrasound (TRUS) showed diffuse inhomogeneity of the left lobe, while only 1 out of the 12 saturation biopsy’s random samples indicated acute prostatitis (in the left lobe), still not justifying the major rise in PSA levels. Due to further increase of the PSA biomarker up to 77 ng/mL, a mpMRI was performed resulting in low probability of clinically significant Pca (PI-RADS 2) (6) but persistence of enlarged pelvic lymph nodes. A [^68^Ga]Ga-PSMA-11 PET/CT was then performed as it allows studying prostate gland, lymph nodes and bones at the same time; it was acquired approximately 60 minutes after the injection of 2 Mbq/Kg of [^68^Ga]Ga-PSMA-11 (according to EAU guidelines (4) and EANM guidelines (7)), with a Field of View (FOV) extended from the vertex to the mid-thigh.

## 3 Diagnostic assessment

**Figure 1:**
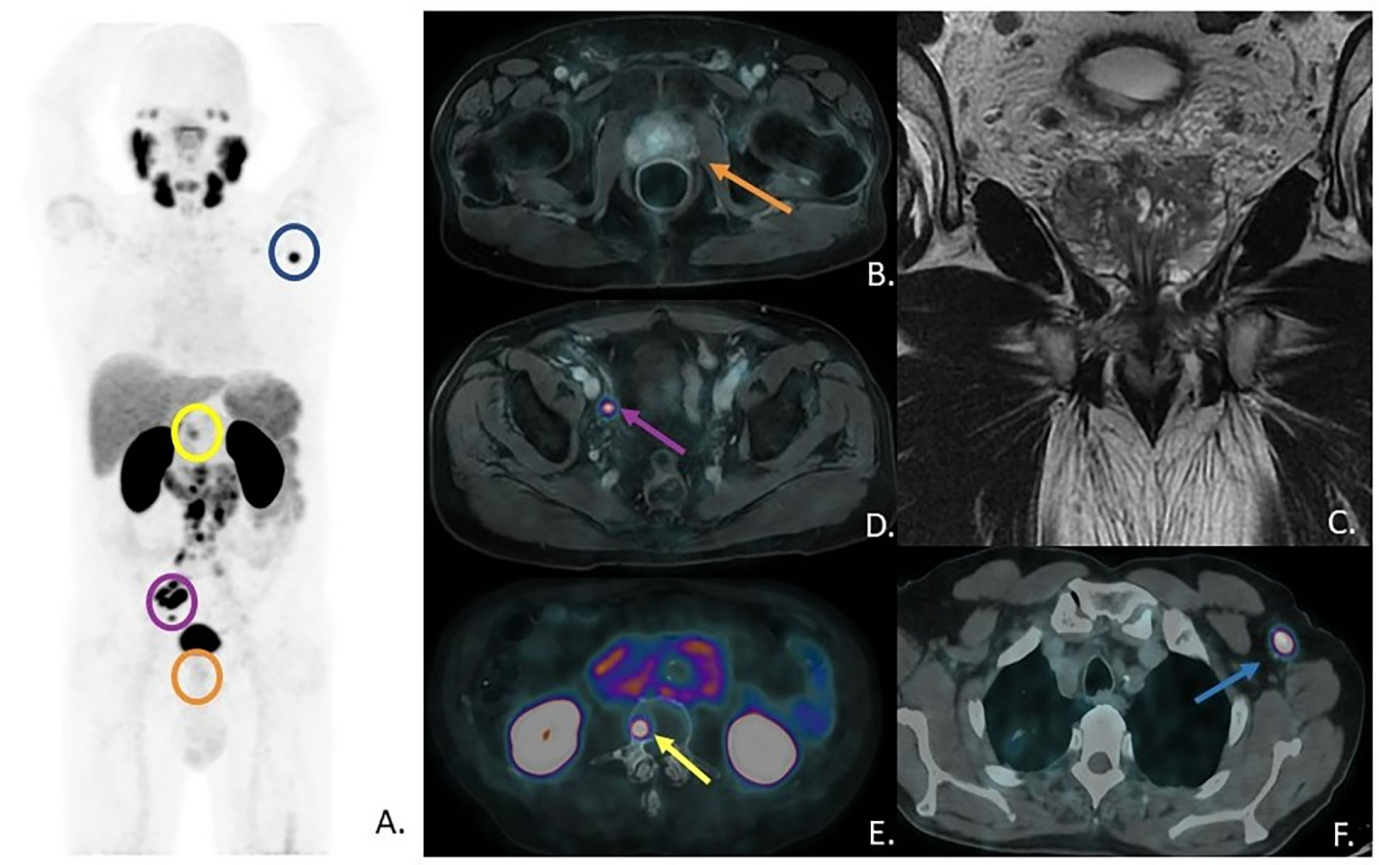
**(A)** [^68^Ga]Ga-PSMA-11 PET Maximum-Intensity-Projection - MIP; **(B)** prostate gland at axial-PSMA-PET fused with previous mpMRI axial T1-Lava sequence; **(C)** prostate gland at mpMRI, coronal view, T2 sequence; **(D)** right external iliac lymph-node uptake at axial PSMA-PET fused with previous mpMRI; **(E)** bone lesion at L3 vertebral body with osteoblastic pattern and multiple lumbar-aortic lymph-nodes at axial fused PSMA-PET/CT; **(F)** left axillary lymph-node uptake at axial fused PSMA-PET/CT.

**Figure 2:**
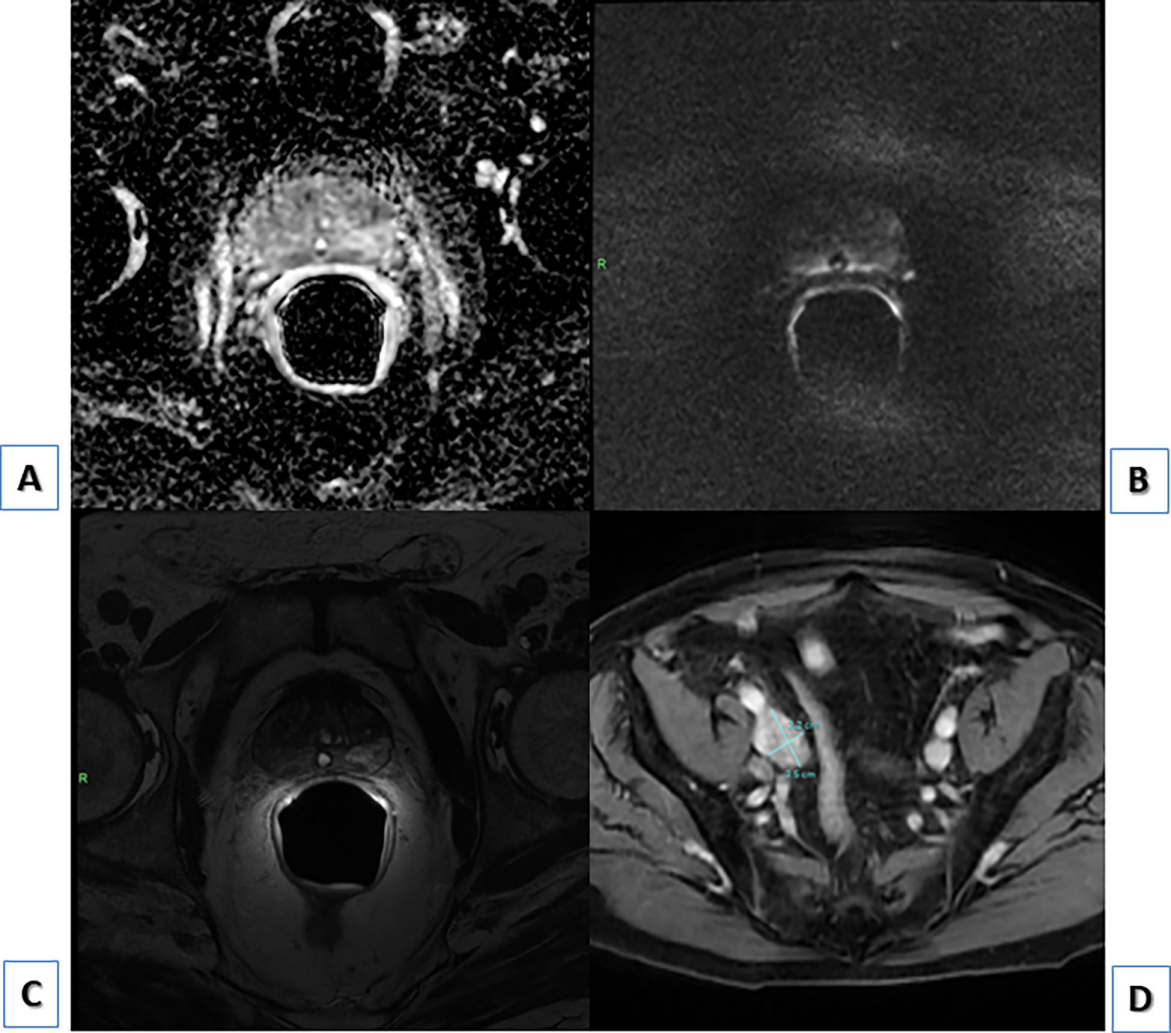
**(A)** PI-RADS 2 prostate gland at mpMRI, ADC, axial; **(B)** PI-RADS 2 prostate gland at mpMRI, DWI, axial; **(C)** PI-RADS 2 prostate gland at mpMRI, T2, axial; **(D)** external iliac enlarged lymph node showing contrast-enhancement at mpMRI, T1 LAVA FLEX sequence, axial.

**Figure 3:**
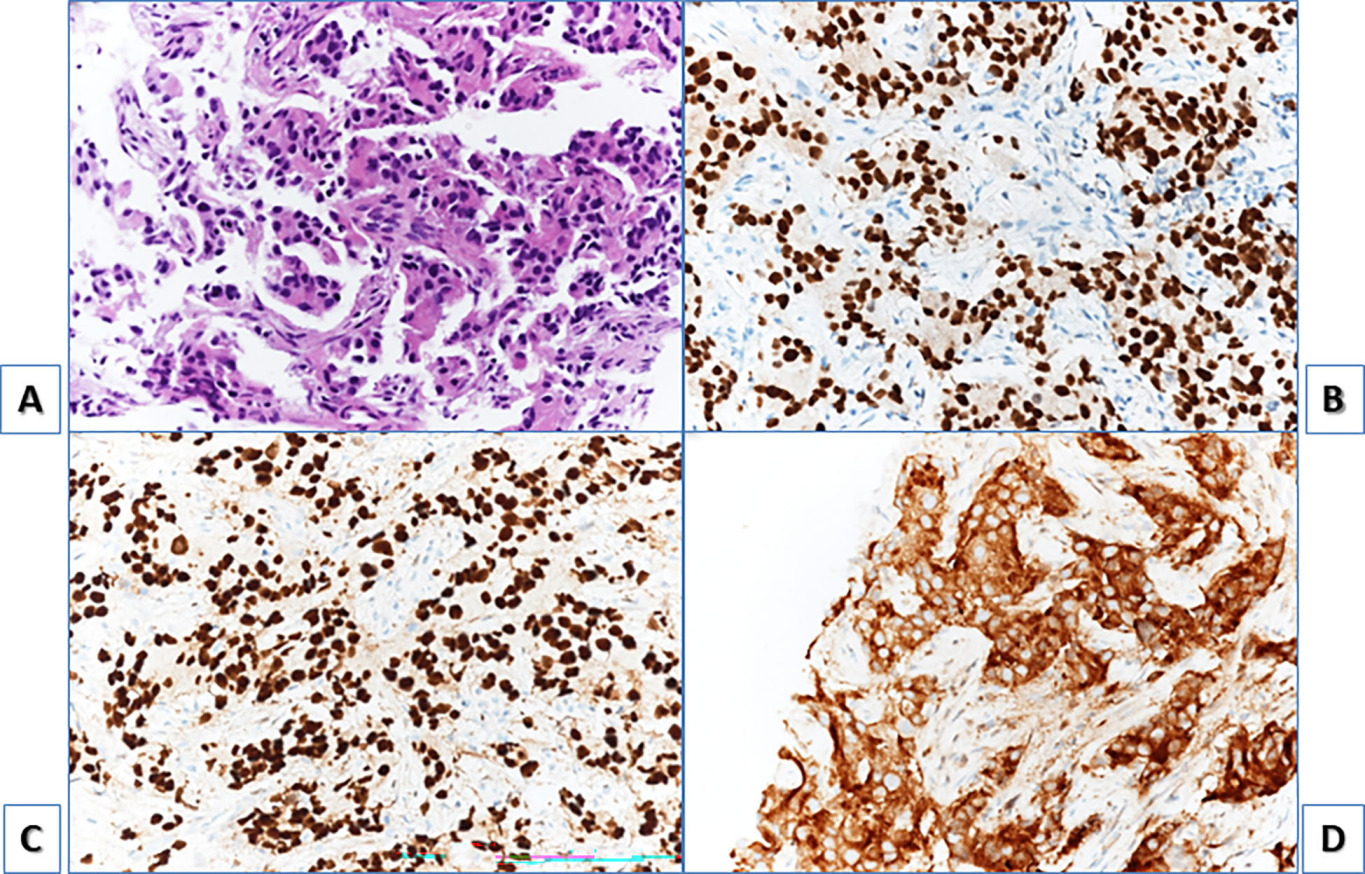
H&E staining, showing axillary lymph node biopsy with neoplastic cells with eosinophilic cytoplasm **(A)**. At immunohistochemistry cells resulted positive for NKX3.1 **(B)** and androgen receptor **(C)** with nuclear positivity for both antibody and for PSA **(D)** cytoplasmatic positivity. They resulted negative for synaptophysin, chromogranin and CK7.

The [^68^Ga]Ga-PSMA-11 PET/CT did not show any significant area of focal uptake within the prostate gland (SUVmax=3.6; (**A,** **Figure 1**) orange circle and (**B**) orange arrow), neither at a 90 minutes-delayed scan; that was in accordance with the previous mpMRI (**C**) showing PI-RADS 2 prostate gland (**Figure 2**). Interestingly, multiple PSMA-avid pelvic and retroperitoneal lymphadenopathies were found (SUVmax=34; (**A,** **Figure 1** **)** purple circle and (**D**) purple arrow, showing an avid right external iliac lymph-node; (**E)** showing multiple lumbar-aortic lymph-nodes). Further findings were also detected: a single, intense focal bone lesion at L3 vertebral body with osteoblastic pattern (SUVmax=14; (**A**) yellow circle, (**E**) yellow arrow) and a single focal uptake in a left axillary lymph-node (SUVmax=19; (**A**) blue circle; (**F**) blue arrow). The axillary lymph-node was homolateral to the injection site of a recent anti-SARS-Cov2 vaccination and morphologically not clearly malignant; however, due to its high PSMA expression, it was therefore biopsied with a mini-invasive ultrasound-guided approach and finally diagnosed as Pca metastasis. Immunohistochemistry was also performed (**Figure 3**). The patient subsequently started Androgen Deprivation Therapy (ADT) plus Docetaxel. Due to adverse drug reaction, therapy was later changed to Cabazitaxel with an initial drop in PSA levels; unfortunately subsequent brain MRI and CT scans detected new multiple brain lesions suspicious for metastases.

## 4 Discussion

Is it known that targeted prostatic biopsy has a few false negative diagnoses and is not immune to flaws (8); this case was particularly challenging as there was concomitant prostate gland negativity at [^68^Ga]Ga-PSMA-11 PET/CT. It is acknowledged that PSMA expression in Pca can show inter- and intra-patient heterogeneity, thus in some cases limiting the use of PSMA scans and directed therapy (9). Our case reflects this concept, not common but well known among clinicians, displaying a heterogeneous scenario in a single patient with absent PSMA expression in the primary tumour but with high PSMA expression in multiple nodes and bone Pca metastasis. Moreover, although other episodes of uncommon locations for Pca presentation are reported in literature, like Virchow’s node or gingival metastasis (10–12), the peculiarity of this case is represented by the metastatic presentation in whom, interestingly, none of the diagnostic techniques were able to detect the primary tumour within the prostate gland. It is well recognized that several malignancies can present with subdiaphragmatic nodal findings and PSMA is not a purely prostate-specific radiotracer, therefore the presence of multiple PSMA avid lymphadenopathies could have also raised the suspicion of lymphoma; however, the osteoblastic bone lesion was more suggestive of Pca. In this particular case [^68^Ga]Ga-PSMA-11 PET/CT was of utmost importance: even if, like MRI, it could not detect the primary prostate cancer it helped to rapidly reach the final diagnosis by detecting extra-pelvic metastasis and selecting the unusual PSMA-avid axillary lesion as the target biopsy, thus optimizing patient’s management; in addition, by detecting a sclerotic bone metastasis typical for PCa (13). This case report is also an example of the crucial importance of using different diagnostic techniques in a multidisciplinary approach when the correct diagnosis is not straightforward.

## Data availability statement

The original contributions presented in the study are included in the article/supplementary material, further inquiries can be directed to the corresponding author/s.

## Ethics statement

This study was performed in line with the principles of the Declaration of Helsinki. The patient gave written consent for the use of anonymous data for research purpose (CE approval: 244/2016/O/Oss). Written informed consent was obtained from the individual for the publication of any potentially identifiable images or data included in this article.

## Author contributions

LV, GC, RM, and LZ contributed to the manuscript conception and design. The first draft of the manuscript was written by LV and GC, and all authors commented on previous versions of the manuscript. All authors contributed to the article and approved the submitted version.
